# Bayesian Estimation for Reliability Engineering: Addressing the Influence of Prior Choice

**DOI:** 10.3390/ijerph18073349

**Published:** 2021-03-24

**Authors:** Leonardo Leoni, Farshad BahooToroody, Saeed Khalaj, Filippo De Carlo, Ahmad BahooToroody, Mohammad Mahdi Abaei

**Affiliations:** 1 Department of Industrial Engineering (DIEF), University of Florence, 50123 Florence, Italy; leonardo.leoni1@stud.unifi.it; 2 Department of Civil Engineering, University of Parsian, Qazvin 3176795591, Iran; farshadbh@gmail.com (F.B.); saeedkhalaj220@gmail.com (S.K.); 3 Marine and Arctic Technology Group, Department of Mechanical Engineering, Aalto University, 11000 Espoo, Finland; ahmad.bahootoroody@aalto.fi; 4 Department of Maritime and Transport Technology, Delft University of Technology, 2628 CD Delft, The Netherlands; M.M.Abaei@tudelft.nl

**Keywords:** reliability analysis, hierarchical Bayesian modelling, prior information, beta-binomial failure modelling

## Abstract

Over the last few decades, reliability analysis has attracted significant interest due to its importance in risk and asset integrity management. Meanwhile, Bayesian inference has proven its advantages over other statistical tools, such as maximum likelihood estimation (MLE) and least square estimation (LSE), in estimating the parameters characterizing failure modelling. Indeed, Bayesian inference can incorporate prior beliefs and information into the analysis, which could partially overcome the lack of data. Accordingly, this paper aims to provide a closed-mathematical representation of Bayesian analysis for reliability assessment of industrial components while investigating the effect of the prior choice on future failures predictions. To this end, hierarchical Bayesian modelling (HBM) was tested on three samples with distinct sizes, while five different prior distributions were considered. Moreover, a beta-binomial distribution was adopted to represent the failure behavior of the considered device. The results show that choosing strong informative priors leads to distinct predictions, even if a larger sample size is considered. The outcome of this research could help maintenance engineers and asset managers in integrating their prior beliefs into the reliability estimation process.

## 1. Introduction

Reliability analysis is of significant importance due to its essential role in dealing with the risk arising from failure events. Indeed, possible failures or accidents may lead to dangerous outcomes [1], resulting in injuries, death, or damage to the environment [2,3,4]. As a result, safety aspects have received a great deal of attention from the public, leading to enterprises’ more stringent reliability requirements [5].

The components usually undergo extensive maintenance actions, which restore the initial operating condition to prevent failures. Any implemented maintenance task produces a cost, mainly composed of three cost items: (i) cost of component, (ii) manpower cost, and (iii) downtime cost. Moreover, when a component is replaced, part of its remaining life is wasted. Therefore, within the development of a maintenance plan, one of the most significant challenges is balancing the cost arising from maintenance interventions and the risk arising from the degraded operating condition. Accordingly, a pivotal role is played by the estimation process of the probabilities of failure. Indeed, estimating accurate probabilities of failure could help to avoid early maintenance without generating safety issues. As a result, there is an ongoing effort in reliability analysis, which has resulted in several methodologies based on different specific assistant tools, including fault-tree (FT) [6,7], fuzzy FT [8,9,10], MLE [11], the first-order reliability method (FORM) [12,13,14] and the second-order reliability method (SORM) [15,16]. Quite recently, Witek [17] presented a three-step approach to estimate the probability of failure when characterizing a high-pressure gas pipeline. In this work, an inspection to assess the corrosion thickness is required, then the probability of failure is computed through a Monte Carlo simulation.

Over the last few decades, Bayesian inference has been exploited by many researchers in several applications, such as maintenance planning [18,19,20], human reliability assessment [21,22,23,24], and reliability analysis [25,26,27,28]. A relevant example of Bayesian network (BN) application for reliability purposes was presented by Abaei et al. [29]. The authors conducted a hydrodynamic analysis to determine the wave load distribution on a floating structure; then, a BN is adopted to estimate the probability of failure. A more recent work by Khalaj et al. [30] displayed the application of BN to assess the reliability of a landslide triggered by an earthquake in a mountainous area. The developed methodology highlighted the displacement of the slope between 1 and 1.5 cm as the most likely. Furthermore, due to the importance of evaluating the operational condition of a component, subsystem, or a system, Bayesian estimation is also widely exploited for failure prognosis and diagnosis [31,32,33]. Zárate et al. [34] proposed a two-phase framework to predict the length of a fatigue crack in structural elements. In the first phase, the authors adopted Bayesian inference to estimate the probability distributions of the fracture mechanism, while during the second phase, the prognosis task was carried out through a Markov chain Monte Carlo (MCMC) process. In another recent work by Sun et al. [35], the Bayesian inference was integrated with least-squares support vector machine to predict the remaining useful life of a microwave component through the specification of a failure threshold.

The attractiveness of BN is related to its key features, which make it one of the most powerful tools for reasoning under uncertainty. Among its strengths, the ability to consider conditional dependencies [36] and the ability to be updated as soon as new information becomes available [37] are worth mentioning. Furthermore, many works have demonstrated the higher efficiency of Bayesian inference compared to other statistical approaches such as MLE [38,39] or FT [40,41]. In a work presented by Li et al. [41], the BN and the FT were exploited for predicting the failure rate of an offshore wind turbine. The results reveal an estimation error equal to 4.5% and 13% for the BN and the FT, respectively, proving the greater accuracy of the BN.

Meanwhile, the improvements in opensource MCMC software, such as OpenBugs, have resulted in wider adoption of the HBM [42], which, compared to the standard BN, provides more robust estimators [43], and it can also cope with source-to-source variability [44]. HBM has been exploited by many researchers for prioritizing maintenance tasks [45,46,47], condition monitoring [48,49], and reliability analysis [50,51]. In a work presented by Andrade and Teixeira [52], the HBM was adopted to evaluate the degradation of the railway between Lisbon and Oporto in Portugal. The proposed methodology considers two quality indicators to determine the evolution of the studied system, providing consistent help in maintenance planning.

Another pivotal property of the Bayesian approach is incorporating into the analysis prior information (e.g., users’ beliefs or expert judgments) for the unknown variables [53], allowing one to deal with limited data. Despite all the ongoing efforts made for enhancing the reliability analysis through Bayesian inference, there is still space to address the influence of the prior choice on the posterior distribution. Consequently, this paper aims at implementing an HBM to conduct the reliability assessment in a closed-mathematical form while considering the impact of the adopted priors on the prediction of future failures. The developed model was verified on three samples characterized by a different number of observations.

### 1.1. Hierarchical Bayesian Modelling

The starting point of any statistical inference is represented by the ‘data’ collected from a stochastic process. By manipulating, evaluating, and organizing data, ‘information’ is obtained, while gathering information leads to acquiring ‘knowledge’. Finally, concluding based on what is known is called ‘inference’ [54]. The HBM is a powerful statistical tool that hinges on Bayes’ Theorem to perform inference [55], as shown by Equation (1).

(1) $$\pi_{1}\left(\theta |x\right)=\frac{f(x|\theta )\pi_{0}\left(\theta \right)}{\int_{\theta }f(x|\theta )\pi_{0}\left(\theta \right)d\theta }$$

where $\theta =\left(\theta_{1},\theta_{2},\ldots \theta_{n}\right)$ is a vector that identifies the unknown parameters of interest (e.g., the mean and the standard deviation characterizing normal distribution). Equation (1) illustrates the proportionality between the posterior distribution, represented by $\pi_{1}\left(\theta |x\right)$, and the product of the likelihood and prior distributions, which are respectively denoted by f(x|θ) and $\pi_{0}\left(\theta \right)$. Hence, the estimated posterior distribution is affected by both the likelihood function (i.e., the data) and the prior distribution. Kelly and Smith [56] stated that the HBM is so named due to the exploitation of multi-stage or hierarchical priors, given by Equation (2).

(2) $$\pi_{0}\left(\theta \right)=\int_{\emptyset }\pi_{1}\left(\theta |\varphi \right)\pi_{2}\left(\varphi \right)d\varphi$$

where *π_1_*(*θ|φ*) is regarded as the first-stage prior, representing the variability of *θ* for a particular value of *φ*, while *π_2_*(*φ*) is the hyper-prior distribution. The hyper-prior distribution considers the variability of *φ*, which is usually a vector whose components are called hyper-parameters.

### 1.2. Prior, Likelihood, Posterior and Predictive Posterior Distribution

The prior, likelihood, and posterior distributions are the essential fundamentals required to conduct Bayesian inference. The prior distribution represents our pre-experimented beliefs on θ. When prior information is included in the Bayesian inference, the prior distribution is regarded as informative. By contrast, the prior distribution is referred to as non-informative when no external information is added to the analysis. The likelihood function specifies the model from which the data are generated. It is the conditional probability of obtaining the data for each possible value of θ, multiplied by a constant factor independent of θ. Finally, the posterior is the Bayesian inference outcome, and denotes our updated knowledge based on prior information and data. It is also worthwhile to introduce the posterior predictive distribution (PPD), representing the prediction of future observations after observing the available data. In other words, given a sample of observations $x=\left(x_{1},x_{2},\ldots x_{n}\right)$, the PPD estimates the probability of obtaining a sample $x^{\prime }=(x_{1}^{\prime },x_{2}^{\prime }\ldots x_{m}^{\prime }$) after the starting data have been analysed, as stated in Equation (3).

(3) $$\pi \left(x^{\prime }|x\right)=\int_{\theta }\pi_{1}\left(x^{\prime }|\theta ,x\right)\pi_{1}\left(\theta |x\right)d\theta$$

Since future observations do not depend on past observations, $\pi_{1}\left(x^{\prime }|\theta ,x\right)$ can be rewritten as $\pi_{1}\left(x^{\prime }|\theta \right)$, which is a likelihood function. Furthermore, $\pi_{1}\left(\theta |x\right)$ identifies the posterior distribution obtained from the Bayesian inference.

### 1.3. Conjugate Prior

A prior distribution is defined as conjugate whenever its combination with the likelihood provides a posterior distribution belonging to the same class of the prior. Let *P* be a class of prior distribution for the unknown parameter θ, and let *S* be a class of sampling distribution f(x|θ). As reported by Gelman et al. [57], *P* is defined as a conjugate for *S* if the following condition is verified:

f(x|θ)∈P   for all   f(·|θ)∈F    and   f(·)∈P

Among the conjugate priors, the most relevant is the so-called natural conjugate prior, characterized by the same functional form of the likelihood. Adopting a natural conjugate prior leads to mathematical convenience; moreover, additional information and beliefs of the user may be incorporated into the prior [58]. Indeed, by choosing a natural conjugate prior, the posterior distribution can be expressed in a closed form.

The remainder of the paper is organized as follows; Section 2 illustrates the proposed approach. Section 3 describes the implementation of the model for the three samples, while Section 4 provides the discussion of the results. Finally, in Section 5, conclusions are drawn.

## 2. Methodology

A binomial distribution was chosen as the likelihood function to model the failure behavior of the considered apparatus. Adopting a binomial likelihood distribution is justified whenever a given device is characterized by a bi-state condition (i.e., failure and safe, usually identified by 0 and 1, respectively). The beta-binomial model aims at estimating the proportion of successes arising from a sequence of Bernoulli trials (or the probability of success for a single trial). In the present work, the probability of success was considered as the probability of failure characterizing the studied equipment (i.e., the probability of obtaining a 0).

At first, three samples characterized by 10, 20, and 40 elements, respectively, were considered, then 5 distinct prior distributions were adopted to assess the impact of the prior choice on different sample sizes. Subsequently, the posterior distributions were found for each prior, and their differences were highlighted. Finally, the PPD was exploited to predict the expected number of failures for each combination of prior and sample size.

### Beta-Binomial Failure Modelling

Let $x_{1},x_{2},\ldots x_{n}$ be a sample of n independent and identically distributed (i.i.d.) observations belonging to a Bernoulli distribution with a probability of success denoted by p, i.e.,:

$$p\in \left[0,1\right]\;x_{i}\;\in \left\{0,1\right\},\;\;x_{i}\sim Bernoulli\left(p\right),\;\;\;i=1,\ldots ,n\;\;i.i.d.$$

Let *k* be the sum of the outcomes of each Bernoulli trial, i.e., the total number of successes:

$$k=\overset{n}{\underset{i=1}{\sum }}x_{i}\;,\;\;\;k\sim Binomial\left(p,n\right)$$

Therefore, the likelihood distribution can be written as illustrated by Equation (4).

(4) $$f(k|p,n)=\left(\begin{array}{c} n\\ k \end{array}\right)p^{k}{\left(1-p\right)}^{n-k}$$

It emerges that the likelihood belongs to the following form:

$$f(k|p)\propto p^{a}{\left(1-p\right)}^{b}$$

Consequently, a natural conjugate prior must be of the same functional form, i.e.,

$$\pi \left(p\right)\propto p^{\alpha -1}{\left(1-p\right)}^{\beta -1}$$

which is the kernel of a beta distribution p~Beta(α,β), whose density is expressed by Equation (5).

(5) $$\pi \left(p\right)=\frac{\Gamma \left(\alpha +\beta \right)}{\Gamma \left(\alpha \right)\Gamma \left(\beta \right)}p^{\alpha -1}{\left(1-p\right)}^{\beta -1}$$

where α and β identify the hyperparameters of the adopted hierarchical model. As stated by Gelman et al. [57], the prior distributions are equivalent to a sample of (α+β−2) Bernoulli trials, where (α−1) successes are observed. The mean and the variance of the beta prior distribution are expressed by Equations (6) and (7), respectively.

(6) $$E\left(p\right)=\frac{\alpha }{\alpha +\beta }$$

(7) $$Var\left(p\right)=\frac{\alpha \beta }{{\left(\alpha +\beta \right)}^{2}\left(\alpha +\beta +1\right)}$$

Accordingly, prior information is inserted into the calculation by adopting specific values for α and β. In reliability analysis, considering high values for α (or low values for β) specifies an unreliable component since the prior mean shifts towards higher values. On the other hand, choosing low values for α (or high values for β) reflects a belief that a particular device is extremely reliable. Moreover, the higher the adopted α and β, the lower the variance, and thus more information is incorporated into the analysis. Indeed, increasing the values of α and β corresponds to a higher number of Bernoulli trials embedded into the prior distribution.

As illustrated by Equation (1), through the application of Bayes’ Theorem, the posterior distribution is obtained:

$$\begin{array}{l} \pi \left(p|k,n\right)\propto f\left(k|p\right)\pi \left(p\right)\\ =\;p^{k}{\left(1-p\right)}^{n-k}p^{\alpha -1}{\left(1-p\right)}^{\beta -1}\\ =\;p^{\alpha +k-1}{\left(1-p\right)}^{\beta +n-k-1} \end{array}$$

which is also a beta density of the form p|k,n~Beta(α+k,β+n−k), thus the posterior distribution is given by Equation (8).

(8) $$\pi \left(p|k,n\right)=\frac{\Gamma \left(\alpha +\beta +n\right)}{\Gamma \left(\alpha +k\right)\Gamma \left(\beta +n-k\right)}p^{\alpha +k-1}{\left(1-p\right)}^{\beta +n-k-1}$$

The posterior mean, illustrated by Equation (9), is usually extracted from the posterior distribution and adopted as a Bayesian estimator for p (i.e., the probability of success for the next Bernoulli trial).

(9) $$E\left(p|k,n\right)=\frac{\alpha +k}{\alpha +\beta +n}$$

Both prior distribution and collected data are represented by the posterior mean, whose value lies between the sample proportion of success k⁄n and the prior mean. As shown by Equation (9), the influence of the prior choice is demoted with large sample sizes, since both k and n increase, and hence E(θ|y) ≈ y/n.

Even if the posterior mean is a good point estimator for the probability of success related to a Bernoulli trial, a more general prediction regarding future observations can be expressed through the PPD. Let n′ be a prospective sample size, then the probability of obtaining precisely k′ successes, for a beta-binomial model, is given by the following PPD:

$$\begin{array}{c} \pi \left(k^{\prime }|k\right)=\int_{0}^{1}\pi \left(k\prime |p,n\prime \right)\pi \left(p|k,n\right)dp\\ =\int_{0}^{1}\frac{n^{\prime }!}{k^{\prime }!\left(n^{\prime }-k^{\prime }\right)!}p^{k^{\prime }}{\left(1-p\right)}^{n^{\prime }-k^{\prime }}\\ X\;\frac{\Gamma \left(\alpha +\beta +n\right)}{\Gamma \left(\alpha +k\right)\Gamma \left(\beta +n-k\right)}p^{\alpha +k-1}{\left(1-p\right)}^{\beta +n-k-1}\\ =\frac{n^{\prime }!}{k^{\prime }!\left(n^{\prime }-k^{\prime }\right)!}\frac{\Gamma \left(\alpha +\beta +n\right)}{\Gamma \left(\alpha +k\right)\Gamma \left(\beta +n-k\right)}\\ X\;\int_{0}^{1}p^{\alpha +k+k^{\prime }-1}{\left(1-p\right)}^{\beta +n-k+n^{\prime }-k^{\prime }-1}dp \end{array}$$

which is a beta-binomial distribution of the form $k^{\prime }|k\sim Beta-binomial\left(n^{\prime },\alpha +k,\beta +n-k\right)$. After solving the integral presented above, the PPD is obtained, as showed by Equation (10).

(10) $$\pi (k^{\prime }|k)=\frac{n^{\prime }!}{k^{\prime }!\left(n^{\prime }-k^{\prime }\right)!}\frac{\Gamma \left(\alpha +\beta +n\right)}{\Gamma \left(\alpha +k\right)\Gamma \left(\beta +n-k\right)}\frac{\Gamma \left(\alpha +k+k^{\prime }\right)\Gamma \left(\beta +n+n^{\prime }-k-k^{\prime }\right)}{\Gamma \left(\alpha +\beta +n+n^{\prime }\right)}$$

The beta-binomial distribution is a binomial distribution with a probability of success which follows a beta distribution with characteristic parameters equal to α+k and β+n−k (i.e., the posterior parameters). Accordingly, the probability of obtaining $k^{\prime }$ successes in $n^{\prime }$ trials is computed by considering the posterior distribution of the probability of success instead of a single value (e.g., the posterior mean).

## 3. Results: Application of the Methodology

To show the applicability of the proposed framework, three samples composed of 10, 20, and 40 observations are considered. From now on, the sample with 10 elements will be referred as the first application, while the sample of 20 and 40 observations will be regarded as the second and the third application, respectively. All the elements in the samples represent distinct components of the same kind. Moreover, the number of failures (i.e., the number of successes or the number of zeros) of each sample is tracked for one year. At the beginning of the year, every component operates in its safe limit condition, and it is regarded as good as new. As shown by Table 1, in the first sample, three failures are observed during the first year of the operations, while in the second sample, six failures occurred during the same time interval. Finally, the third sample is characterized by 12 failures in one year.

### 3.1. Prior Choice

The five different prior distributions chosen for this study are illustrated in Table 2. The adopted hyper-parameters were chosen to include in the study a prior distribution concealing no information, two prior distributions reflecting a belief of unreliability with a distinct level of information, and two prior distributions identifying a reliable component with a distinct level of information once again.

BPD 1 is regarded as non-informative since it is equivalent to a uniform distribution of the form p~Unif(0,1), hence all the values of the probability of failure are equally likely. The prior sample size is given by (α+β−2); therefore, p~Beta(1,1) is interpreted as a zero-dimensional sample. BPD2 reflects a strong belief that the components are very reliable since it represents a prior sample with one failure in nine observations. On the other side, BPD 3 conceals a piece of robust information regarding the unreliability of the considered device. Indeed, it can be seen as a sample of nine elements characterized by 4eight failures. Finally, BPD 4 and BPD 5 denote a high- and a low-reliability component, respectively. Compared to the previous two distributions, less information is included in the fourth and the fifth prior, since they both are interpreted as a sample composed of two observations.

### 3.2. Posterior Distribution

**Figure 1:**
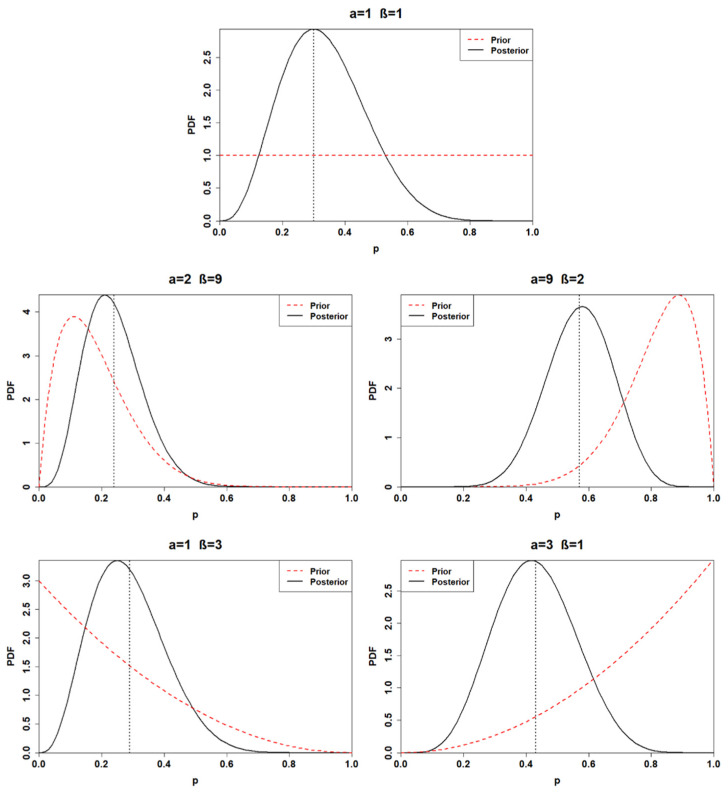
Posterior distributions of the first application (sample with *n* = 10, *k* = 3) for distinct choices of prior. The black dashed lines represent the posterior mean values.

Adopting a beta conjugate prior for a binomial likelihood function, the posterior distribution can be expressed as p|k,n~Beta(α+k,β+n−k). Thus, the posterior distribution parameters are obtained through a combination of the data and the hyperparameters. Accordingly, the posterior distributions for the first application are found. From now on, each posterior distribution will be associated with a number corresponding to the prior distribution from which the posterior is generated (e.g., posterior 1 is obtained through the adoption of BPD 1). Table 3 and Figure 1 show the posterior distributions obtained from each prior choice.

A minimal difference is recognized between the first and the fourth posterior distribution. Indeed, the first posterior mean is estimated at 0.33, while the fourth is 0.29. Furthermore, the first and the fourth posterior distribution yields a 95% credible interval of (0.11, 0.61) and (0.09, 0.53), respectively. As depicted by Figure 1, the posterior distribution is strongly prior-driven, in the cases when BPD 2 and BPD 3 are adopted. The third posterior mean is computed at 0.57, which can be interpreted as predicting six failures in 10 observations. On the other hand, the second posterior yields a mean of 0.24, denoting a much more reliable component. Finally, the fifth posterior distribution lies between the third and the fourth, with a mean equal to 0.43 and a 95% credible interval (0.19, 0.68).

**Figure 2:**
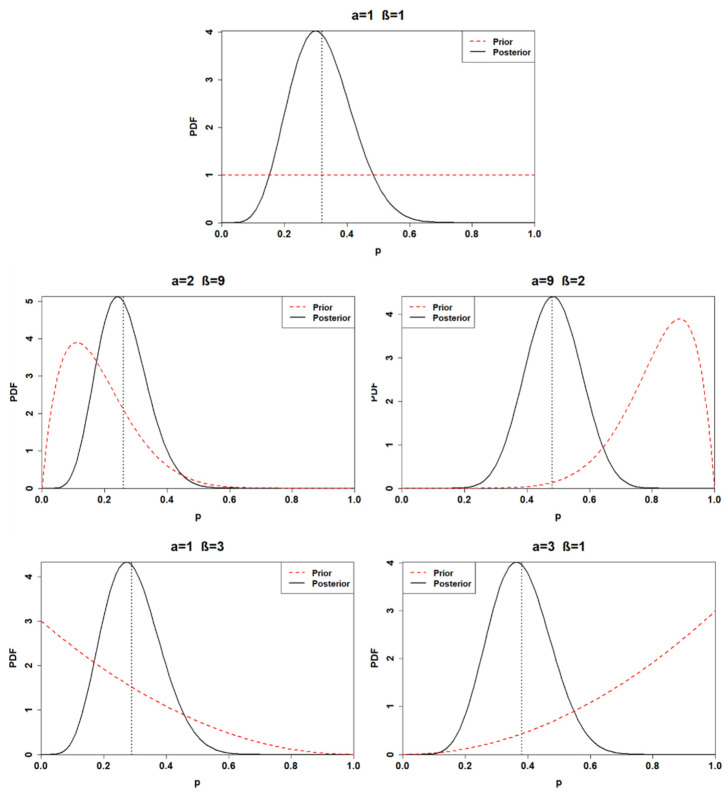
Posterior distributions of the second application (sample with *n* = 20, *k* = 6) for distinct choices of prior. The black dashed lines represent the posterior mean values.

As previously executed for the first application, the posterior distributions are also calculated for the second one. The results are illustrated in Table 4 and Figure 2.

The posterior distributions of the second application are characterized by less uncertainty than the posterior distributions calculated for the first application. Indeed, comparing, for instance, the first posterior of the first application and the first posterior of the second application, the calculation revealed smaller 95% credible intervals associated with the posterior of the second application. The same results are obtained for all the other posterior distributions as well. The first and the fourth posterior distributions show remarkable similarities once again, having a 95% posterior credible interval of (0.14, 0.52) and (0.13, 0.48), respectively. The third posterior distribution is still deeply influenced by the prior; indeed, its mean is located at 0.48. Finally, the second and the fifth prior yield a mean of 0.26 and 0.38, respectively.

**Figure 3:**
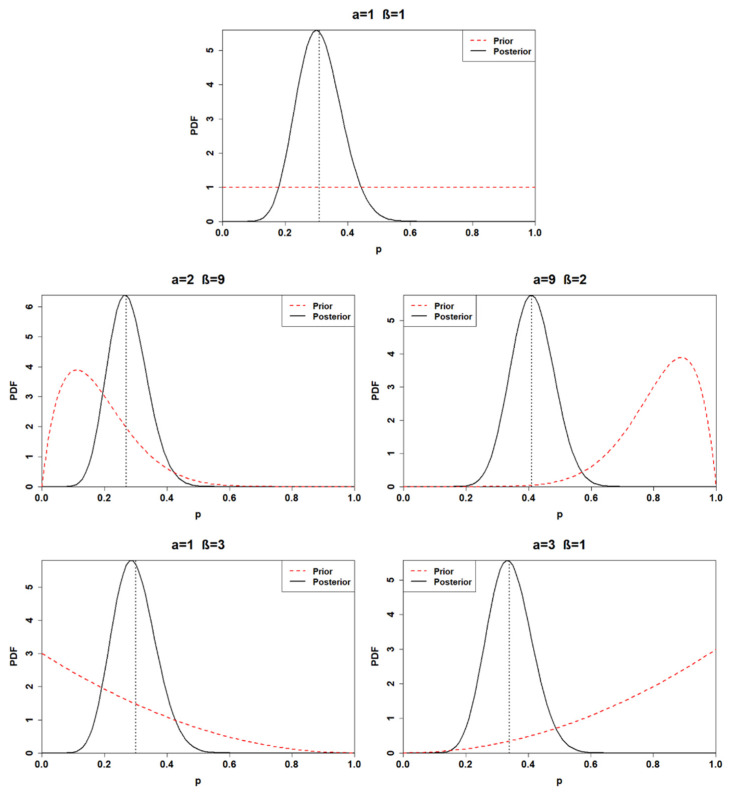
Posterior distributions of the third application (sample with *n* = 40, *k* = 12) for distinct choices of prior. The black dashed lines represent the posterior mean values.

Finally, the calculation process is replicated for the posterior distribution related to the third application. The parameters of each posterior distribution are listed in Table 5, while the obtained posterior distributions are plotted in Figure 3.

The uncertainty of the posterior distributions is reduced compared to the previous two applications; moreover, the influence of the prior choice is demoted. Indeed, the means of the first, the second, the fourth, and the fifth posterior distribution are included between 0.27 and 0.34. The only distribution characterized by a mean higher than 0.4 is the third posterior, which has a 95% credible interval of (0.28, 0.55).

### 3.3. Predictive Posterior Distribution

**Figure 4:**
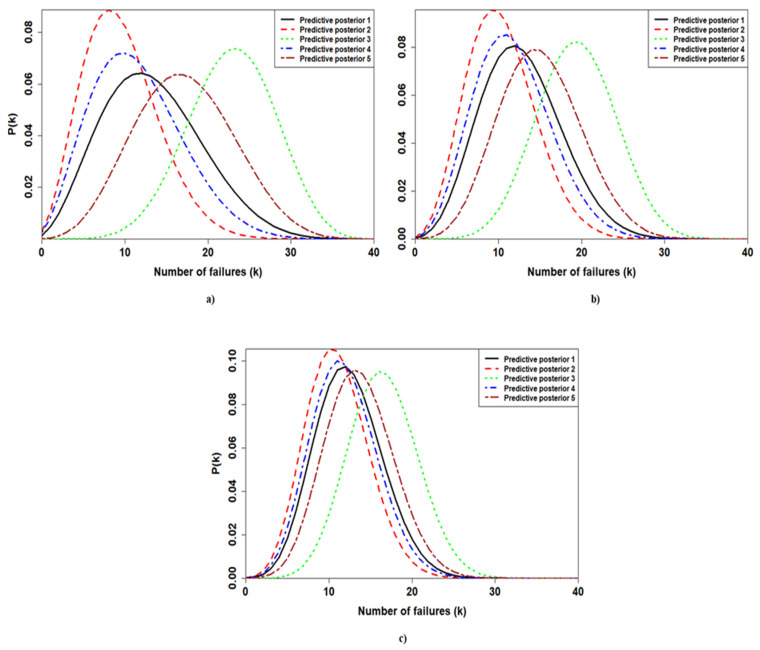
Beta-binomial posterior predictive distributions (PPDs) for distinct priors. (**a**–**c**) arise from the first application (the sample with *n* = 10, *k* = 3), the second application (the sample with *n* = 20, *k* = 6), and the third application (the sample with *n* = 40, *k* = 12), respectively. The number associated with each PPD is the same as the prior from which the PPD is generated.

The beta-binomial posterior distributions are developed for each application to make predictions about the future number of failures (Figure 4). As a future sample size, 40 observations were chosen.

As depicted by Figure 4, varying the prior results in a striking difference among the obtained PPDs for the first application. By contrast, the PPDs associated with the third application shrink by similar values. The only PPD which falls further than the others is the third one. Finally, the PPDs of the second application are less dispersed than the PPDs of the first application. However, they show fewer similarities compared to the PPDs estimated for the third application.

## 4. Discussion

**Figure 5:**
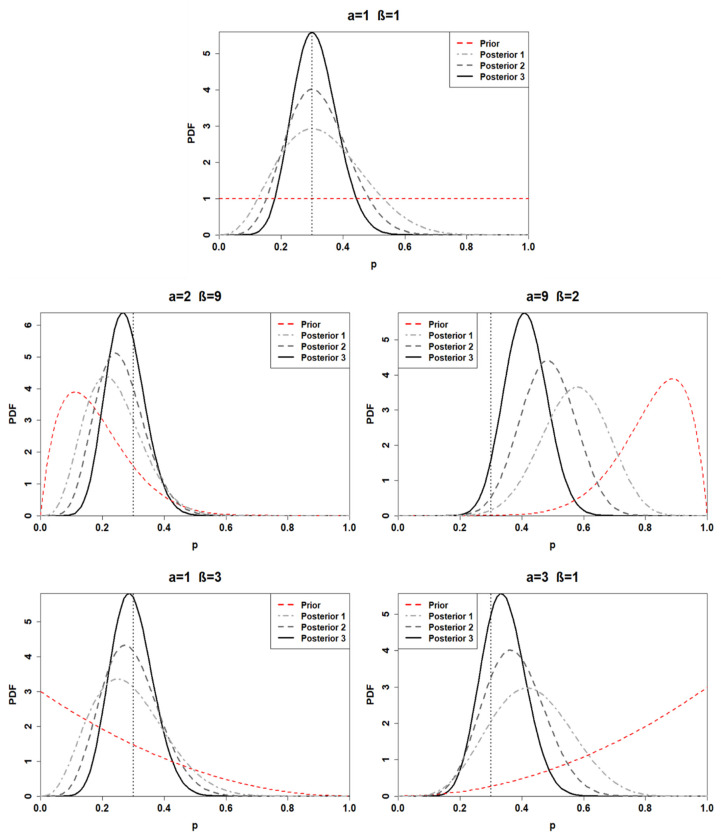
Developed posterior distributions for each choice of prior, which is identified by the values of α and β above each graph. Posterior 1, Posterior 2, and Posterior 3 denote the posterior distributions obtained for the first, second, and third applications. At last, the vertical dotted line represents the likelihood means.

Given a certain prior, Figure 5 compares the obtained posterior distributions for distinct applications. Figure 5 can be seen as a summary of Figure 1, Figure 2 and Figure 3.

**Figure 6:**
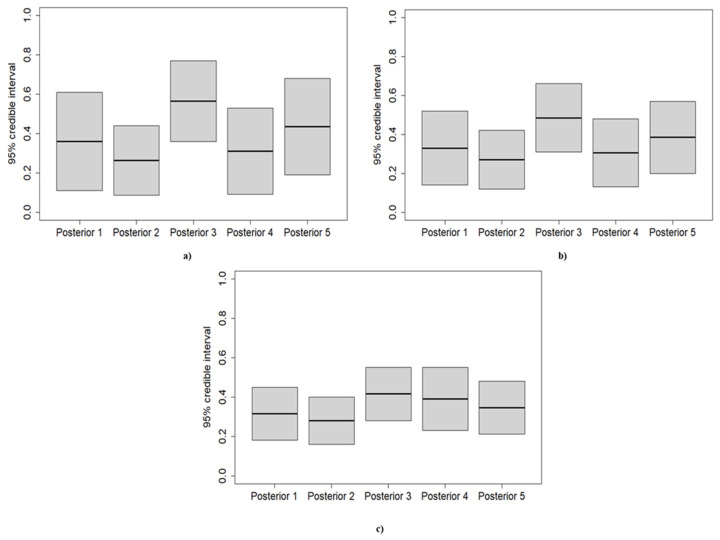
Uncertainties represented by the 95% credible interval of each posterior distribution for distinct priors. (**a**–**c**) arise from the first application (the sample with *n* = 10, *k* = 3), the second application (the sample with *n* = 20, *k* = 6), and the third application (the sample with *n* = 40, *k* = 12), respectively.

As depicted by Figure 5, the posterior distributions shift towards the likelihood for greater sample size, identifying a weaker influence of the adopted prior on the posterior distribution. Moreover, the variance of the posterior distribution decreases for the samples characterized by a higher number of observations, denoting less uncertainty (Figure 6)

**Figure 7:**
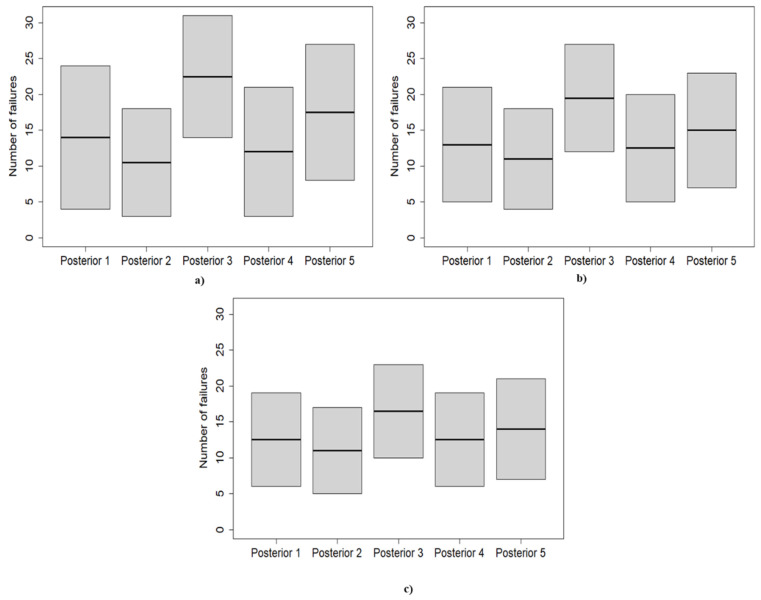
Boxplot of the expected number of failures corresponding to a 0.90 confidence interval. (**a**–**c**) refers to the PPDs calculated for the first, second, and third applications, respectively.

Finally, to underline how the prior choice affects the reliability analysis and the subsequent maintenance planning, the inverse cumulative PPDs are exploited to predict the expected number of failures during the next year of operation. Adopting cumulative probabilities of 0.05 and 0.95, the corresponding number of failures was calculated. The results are presented in Table 6 and Figure 7.

Considering the first application, the calculation revealed significant variability in the expected number of failures for different prior choices. Adopting BPD 3, the inverse beta-binomial predicted a number of failures between 14 and 31 with a confidence interval of 0.90, while choosing BPD 2 results in expecting the component to fail between 3 and 18 times with the same confidence interval. Thus, the adoption of BPD 3 leads one to schedule more maintenance actions during the next year of operations, and consequently higher costs related to the purchase and the management of the spare parts are accounted for. By contrast, the exploitation of the BPD 2 predicts few maintenance efforts; therefore, the cost associated with maintenance activities is lower; however, this could result in a higher risk to the operations. Selecting BPD 1 and BPD 4 provides similar results for the PPDs, predicting the device to fail between 4 and 24 times and 3 and 21 times, respectively. Finally, picking the BPD 5 produces an expected number of failures between 8 and 27 with a 0.90 confidence interval.

For the second application, the component is foreseen to fail between 12 and 27 times with a 0.90 confidence interval if BPD 3 is adopted. When BPD 1 is chosen, between 5 and 21 failures are predicted. A similar result is provided by adopting BPD 4, which predicts between 5 and 20 failures. The second PPD predicts few maintenance efforts; indeed, the equipment is expected to have between 4 and 18 maintenance actions. Finally, choosing BPD 5 results in predicting between 7 and 23 failures during the next year.

As highlighted by Table 6 and Figure 7, the third sample is characterized by the lowest PPD-to-PPD variability; thus, the calculation is mostly data-driven. Indeed, considering a 0.90 confidence interval, between 6 and 19 failures are predicted by both the first and the fourth PPDs. Similar results are also produced by the second and the fifth PPD. Between 4 and 17 failures are foreseen by the second PPD, while the adoption of the BPD 5 results in expecting the component to fail between 7 and 21 times. However, the third PPD, which arises from a strong informative prior, is quite detached from the others, and it is characterized by an expected number of failures between 10 and 23. Consequently, considering the third PPD leads to much more maintenance effort than the other PPDs, resulting in a different maintenance plan.

Considering the obtained predictions, it is possible to state that the decision-making process is influenced by the prior choice, even if many samples of observations are available. Thus, going fully informative (for instance, BPD 3) could determine inaccurate predictions, leading to improper maintenance strategies. On the other hand, adopting weakly informative priors allows one to insert prior information while letting the data speak for themselves. For instance, BPD 5 denotes a belief of unreliability, but the data are still able to influence the posterior distribution strongly. By contrast, the BPD 3 overcomes the data even in the case of a large sample, having a considerable impact on the predictions.

## 5. Conclusions

One of the major advantages of Bayesian Inference over other statistical tools is the ability to consider prior information. Within the reliability analysis process, this feature can be exploited to insert users’ beliefs regarding the reliability of a given device. However, incorporating information into the analysis could deeply affect the reliability calculation and the subsequent decision-making process regarding maintenance schedules. In this paper, a mathematical application of Bayesian inference for reliability analysis is presented. Furthermore, the influence of the prior choice on the reliability estimation process is investigated. This task was carried out for three simple applications, considering a beta-binomial failure modelling. The results depict that for greater sample sizes, less influence of the prior choice and less uncertainty is presented by the posterior distributions. Nevertheless, the posterior distributions and the predictions regarding the future number of failures are still affected to a certain degree by particularly informative priors. As a result, adopting strong informative priors is not always a wise choice, because the subsequent decisions could be based on misleading information. Accordingly, the adoption of weakly informative priors is strongly recommended in cases where few data are observed, while non-informative priors should be exploited when sufficient data are available. It is worthwhile mentioning that available prior knowledge could be enhanced through simulation processes. As a further development, distinct failure modelling could be considered to address how the prior choice affects the posterior distributions. Moreover, multi-state components (e.g., normal, degraded and fault) could be studied by grouping the distinct states into two appropriate outcomes (e.g., normal and degraded could be grouped as working), which allows one to repeat the binomial experiment. After finding the probability distributions for the grouped outcomes, they could be split again to find the probability distribution of the original single outcomes. To study multi-state components, a multinomial distribution could also be adopted. Indeed, multinomial distribution is the natural extension of binomial distribution, and it can consider more than two outcomes for each trial.

## Figures and Tables

**Table 1:** Adopted samples with their respective observations and observed failures.

Sample	# Observations (*n*)	# Failures (*k*)
Sample 1	10	3
Sample 2	20	6
Sample 3	40	12

**Table 2:** Parameters of the adopted beta prior distributions.

Beta Prior Distribution (BPD)	Hyper-Parameters		Prior Mean	Prior Variance
	Alpha	Beta		
BPD 1 (non-informative)	1	1	0.5	0.08
BPD 2 (very reliable)	2	9	0.18	0.01
BPD 3 (very unreliable)	9	2	0.82	0.01
BPD 4 (quite reliable)	1	3	0.25	0.04
BPD 5 (quite unreliable)	3	1	0.75	0.04

**Table 3:** Parameters of each posterior distribution for the first application (sample with *n* = 10, *k* = 3).

# Posterior	Alpha	Beta	Posterior Mean	95% Posterior Interval for *p*
Posterior1	4	8	0.33	[0.11, 0.61]
Posterior2	5	16	0.24	[0.086, 0.44]
Posterior3	12	9	0.57	[0.36, 0.77]
Posterior4	4	10	0.29	[0.09, 0.53]
Posterior5	6	8	0.43	[0.19, 0.68]

**Table 4:** Parameters of each posterior distribution for the second application (sample with *n* = 20, *k* = 6).

# Posterior	Alpha	Beta	Posterior Mean	95% Posterior Interval for *p*
Posterior1	7	15	0.32	[0.14, 0.52]
Posterior2	8	23	0.26	[0.12, 0.42]
Posterior3	15	16	0.48	[0.31, 0.66]
Posterior4	7	17	0.29	[0.13, 0.48]
Posterior5	9	15	0.38	[0.20, 0.57]

**Table 5:** Parameters of each posterior distribution for the third application (sample with *n* = 40, *k* = 12).

# Posterior	Alpha	Beta	Posterior Mean	95% Posterior Interval for *p*
Posterior1	13	29	0.31	[0.18, 0.45]
Posterior2	14	37	0.27	[0.16, 0.40]
Posterior3	21	30	0.41	[0.28, 0.55]
Posterior4	13	31	0.30	[0.23, 0.55]
Posterior5	15	29	0.34	[0.21, 0.48]

**Table 6:** Number of failures corresponding to a cumulative probability of 0.05 and 0.95 (considering 40 observations in the future sample).

	First Application		Second Application		Third Application	
# Posterior	0.05	0.95	0.05	0.95	0.05	0.95
Posterior 1	4	24	5	21	6	19
Posterior 2	3	18	4	18	5	17
Posterior 3	14	31	12	27	10	23
Posterior 4	3	21	5	20	6	19
Posterior 5	8	27	7	23	7	21

## Data Availability

No new data were created or analyzed in this study. Data sharing is not applicable to this article.
